# *BMP4* Was Associated with NSCL/P in an Asian Population

**DOI:** 10.1371/journal.pone.0035347

**Published:** 2012-04-13

**Authors:** Qianqian Chen, Hong Wang, Jacqueline B. Hetmanski, Tianxiao Zhang, Ingo Ruczinski, Holger Schwender, Kung Yee Liang, M. Daniele Fallin, Richard J. Redett, Gerald V. Raymond, Yah-Huei Wu Chou, Philip Kuo-Ting Chen, Vincent Yeow, Samuel S. Chong, Felicia S. H. Cheah, Ethylin Wang Jabs, Alan F. Scott, Terri H. Beaty

**Affiliations:** 1 Department of Epidemiology and Biostatistics, School of Public Health, Peking University, Beijing, China; 2 Department of Epidemiology, Johns Hopkins Bloomberg School of Public Health, Baltimore, Maryland, United States of America; 3 Division of Biology and Biomedical Sciences, Washington University, St. Louis, Missouri, United States of America; 4 Department of Faculty of Statistics, TU Dortmund University, Dortmund, Germany; 5 Department of Life Sciences and Institute of Genome Sciences, National Yang-Ming University, Taipei, Taiwan; 6 Department of Surgery, Johns Hopkins University School of Medicine, Baltimore, Maryland, United States of America; 7 Kennedy Krieger Institute, Johns Hopkins University School of Medicine, Baltimore, Maryland, United States of America; 8 Department of Medical Research, Chang Gung Memorial Hospital, Taipei, Taiwan; 9 Craniofacial Center, Chang Gung Memorial Hospital, Taipei, Taiwan; 10 Department of Plastic Surgery, K K Women's and Children's Hospital, Singapore, Singapore; 11 Department of Pediatrics, National University of Singapore, Singapore, Singapore; 12 Department of Pediatrics, Johns Hopkins University School of Medicine, Baltimore, Maryland, United States of America; 13 Department of Genetics and Genomic Sciences, Mount Sinai School of Medicine, New York City, New York, United States of America; 14 Department of Medicine, Johns Hopkins University School of Medicine, Baltimore, Maryland, United States of America; University of Ottawa, Canada

## Abstract

**Background:**

The Bone Morphogenetic Protein 4 gene (*BMP4*) is located in chromosome 14q22-q23 which has shown evidence of linkage for isolated nonsyndromic cleft lip with or without cleft palate (NSCL/P) in a genome wide linkage analysis of human multiplex families. *BMP4* has been shown to play crucial roles in lip and palatal development in animal models. Several candidate gene association analyses also supported its potential risk for NSCL/P, however, results across these association studies have been inconsistent. The aim of the current study was to test for possible association between markers in and around the *BMP4* gene and NSCL/P in Asian and Maryland trios.

**Methodology/Principal Findings:**

Family Based Association Test was used to test for deviation from Mendelian assortment for 12 SNPs in and around *BMP4*. Nominal significant evidence of linkage and association was seen for three SNPs (*rs10130587*, *rs2738265* and *rs2761887*) in 221 Asian trios and for one SNP (*rs762642*) in 76 Maryland trios. Statistical significance still held for *rs10130587* after Bonferroni correction (corrected p = 0.019) among the Asian group. Estimated odds ratio for carrying the apparent high risk allele at this SNP was 1.61 (95%CI = 1.20, 2.18).

**Conclusions:**

Our results provided further evidence of association between *BMP4* and NSCL/P.

## Introduction

Cleft lip with or without cleft palate (CL/P) is the most common human craniofacial malformation with a prevalence in newborns between 1/500–1/2000 worldwide [1], [2], accounting for approximately one third of all congenital abnormalities [3]. In etiologic studies, CL/P is usually classified as syndromic or nonsyndromic based on the concurrent existence of other congenital malformations or developmental abnormalities. Almost 70% of the children born with a CL/P phenotype are nonsyndromic cases [4]–[7]. As the lip and primary palate have distinct developmental origins from the secondary palate, clefts of these areas can be further subdivided into cleft lip with or without cleft palate and cleft palate alone to maximize homogeneity of cleft phenotype [8]. Although a large number of candidate gene studies have been conducted in this field, and several genome-wide association studies (GWAS) [9]–[12] have recently provided more clues, specific causal variants and biological mechanisms responsible for occurrence of nonsyndromic cleft lip with or without cleft palate (NSCL/P) remain unclear.

The bone morphogenetic protein 4 gene (*BMP4*) on chromosome 14q22-q23 is one of the promising candidate genes for NSCL/P. Animal models show it is predominately expressed in epithelia of the maxillary and mandibular primordia [13], [14], as well as the mesenchymal tissue around the frontonasal process and lateral maxillary prominence in the *C578L/6J* mice [15]. When the exogenous antagonist Noggin for BMP2 and BMP4 was administered to the region of beak fusion, visible clefts of the upper beak were shown in 10 of 13 chick embryos [14]. Further, bilateral fusion delay of the lip was reported in all nine mice with *BMP4* conditional null allele at 12 embryonic days [16]. In humans, the chromosomal region around 14q22-q23 gave evidence of linkage (significant at a genome-wide level) to a gene controlling NSCL/P in multiplex NSCL/P families recruited from various populations [17], [18]. However, results from published association studies have been inconsistent [9]–[12], [19]–[22] suggesting a need for further research.

Here we tested for linkage and association between markers in and around *BMP4* gene and NSCL/P using 297 case parent trios from Maryland and three Asian populations to further clarify the potential role *BMP4* may play in the etiology of this common and complex disorder. Our results provided further evidence of association between *BMP4* and NSCL/P.

## Materials and Methods

### Ethics statement

Study protocols were reviewed and approved by the institutional review boards in all participating institutions. All adult participants (including adult probands and probands' parents) and parents or guardians of pediatric participants provided written informed consent.

### Sample description

A total of 297 NSCL/P probands and their parents were recruited from four cleft lip and palate treatment centers: Maryland (76 trios from Johns Hopkins and University of Maryland hospitals), Taiwan (146 trios from Chang Gung Memorial Hospital), Singapore (35 trios from KK Women's and Children's Hospital), and Korea (40 trios from Yonsei Medical Center) (Table 1). All probands had received clinical genetic assessment to check for other birth defects or developmental delays and were all diagnosed as NSCL/P cases.

**Table 1:** Gender of NSCL/P probands.

Site	Male	Female	Total
Maryland	44	32	76
Korea	22	18	40
Singapore	24	11	35
Taiwan	95	51	146
Total	185	112	297

### SNP selection, DNA, and genotyping

**Figure 1:**
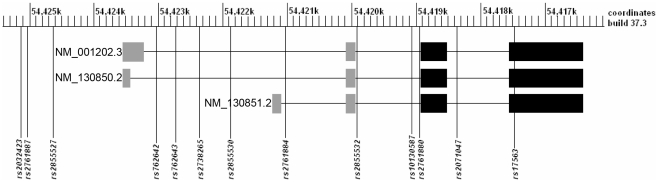
Schematic genomic structure of *BMP4* and coordinates of 13 genotyped SNPs. Three variants (NM_001202.3, NM_130850.2 and NM_130851.2) of the human *BMP4* gene are shown in the figure with 4 exons each aligned from left to right. Filled gray and black boxes represent the untranslated and translated exons respectively. All three variants encode an identical protein through translation of exons 3 and 4. Scale on top of the figure shows position of the gene and coordinates for the 13 genotyped SNPs basing on GRCh build 37.3. Minor allele frequency for one SNP (*rs2855527*) was less than 1% in both Asian and Maryland founders and was excluded from association analysis.

A total of 13 SNPs (Table 2 and 3, and Fig. 1) were genotyped in and around *BMP4* with a goal of identifying an average of one SNP per 5 kilobase pairs (kb) of physical distance where one SNP locates in exon 4 (*rs17563*), another nine SNPs locate in introns and the additional 3 SNPs reside in the 5′ untranslated region of the gene basing on variant NM_130850.2 or NM_001202.3 (http://www.ncbi.nlm.nih.gov/gene/652).

**Table 2:** TDT analysis for 12 SNPs in and around *BMP4* in Asian trios.

SNP name	Position	Minor	MAF	FAM*	T	NT	OR (95%CI)	*P* Value	Bonferroni corrected *P* Value
		allele	(%)						
*rs17563*	54417522	2	25.4	122	77	72	1.07 (0.78,1.48)	0.6877	1.0000
*rs2071047*	54418411	1	45.9	136	81	98	0.83 (0.62,1.11)	0.2045	0.9358
*rs2761880*	54418986	1	26.7	119	79	66	1.20 (0.86,1.66)	0.2842	0.9819
*rs10130587*	54419110	2	48.7	139	113	70	1.61 (1.20,2.18)	0.0016	0.0190
*rs2855532*	54419965	1	43.0	145	104	82	1.27 (0.95,1.69)	0.1082	0.7469
*rs2761884*	54421052	1	42.4	153	113	88	1.28 (0.97,1.70)	0.0769	0.6172
*rs2855530*	54421917	1	49.6	148	110	92	1.20 (0.91,1.58)	0.2083	0.9394
*rs2738265*	54422399	2	42.3	148	112	84	1.33 (1.01,1.77)	0.0489	0.4521
*rs762643*	54422767	1	43.1	108	75	67	1.12 (0.81,1.56)	0.5020	0.9998
*rs762642*	54423053	2	43.3	141	84	104	0.81 (0.61,1.08)	0.1449	0.8472
*rs2761887*	54425052	2	43.1	150	115	84	1.37 (1.03,1.81)	0.0283	0.2914
*rs2032423*	54425147	1	43.0	151	113	87	1.30 (0.98,1.72)	0.0683	0.5721

FAM*: number of informative families, T: number of transmitted alleles, NT: number of un-transmitted alleles.

**Table 3:** TDT analysis for 12 SNPs in and around *BMP4* in Maryland trios.

SNP name	Position	Minor allele	MAF (%)	FAM*	T	NT	OR (95%CI)	*P* Value	relative difference ratio
*rs17563*	54417522	1	47.7	36	26	24	1.08 (0.62, 1.89)	0.7789	-
*s2071047*	54418411	1	43.5	37	25	24	1.04 (0.60, 1.82)	0.8817	5.23
*rs2761880*	54418986	1	3.9	5	4	2	2.00 (0.37, 10.92)	0.4178	85.39
*rs10130587*	54419110	1	40.8	33	23	22	1.05 (0.58, 1.88)	0.8837	-
*rs2855532*	54419965	1	46.1	38	19	33	0.58 (0.33, 1.01)	0.0515	−7.21
*rs2761884*	54421052	1	43.7	38	22	31	0.71 (0.41, 1.23)	0.2188	−3.07
*rs2855530*	54421917	2	48.1	41	35	24	1.46 (0.87, 2.45)	0.1530	-
*rs2738265*	54422399	2	45.1	35	20	29	0.69 (0.39, 1.22)	0.1954	−6.62
*rs762643*	54422767	1	41.0	35	18	30	0.60 (0.33, 1.08)	0.0833	−1.41
*rs762642*	54423053	2	39.3	39	36	21	1.71 (1.00, 2.94)	0.0469	9.24
*rs2761887*	54425052	2	44.7	33	18	28	0.64 (0.36, 1.16)	0.1442	−3.71
*rs2032423*	54425147	1	45.1	37	22	28	0.79 (0.45, 1.37)	0.3994	−4.88

FAM*: number of informative families.

T: number of transmitted alleles, NT: number of un-transmitted alleles.

Relative difference ratio: Relative difference ratio for MAFs between Asian and Maryland parents.

SNPs with “SNP scores" >0.6 (an assessment of design quality of the Illumina assay based on a proprietary algorithm), high validation levels in dbSNP (including validation on multiple platforms), and high heterozygosity levels were given priority when selecting SNPs. All 3 SNPs released by the HapMap project (HapMap Data Rel 24/phaseII Nov08, on NCBI B36 assembly, dbSNP b126) were included in our marker panel. The genomic DNA was extracted from peripheral blood using the protein precipitation method and stored at −20°C. Aliquots (4 µg) of each genomic DNA sample were dispensed into a bar-coded 96-well microtiter plate at a concentration of 100 ng/µl and genotyped by Illumina's GoldenGate chemistry [23] at the Genetic Resources Core Facility (GRCF) of Johns Hopkins University. Two duplicates and four controls from the Centre d'Etude du Polymorphisme Humain (CEPH) collection were included on each plate to evaluate genotyping consistency within and between plates.

### Statistical analysis

#### (1) SNP screening and preliminary analysis

We first screened SNPs for minor allele frequency (MAF), pairwise linkage disequilibrium (LD) measured as *r^2^*, and Hardy-Weinberg equilibrium (HWE) for parents within each population (and in three Asian populations combined) using Haploview (v4.2, http://www.broadinstitute.org/haploview/haploview) [24]. SNPs with MAF>1%, genotyping call rate >80% in each group, and showing adherence to HWE at p>0.01 were eligible for analysis. The relative difference ratio was calculated as the difference in MAFs for parents in Maryland and the combined Asian samples, divided by the MAF in Asian parents for each SNP.

#### (2) Association analysis

We performed transmission disequilibrium test (TDT) analysis, originally proposed by Spielman et al. [25] on each eligible marker, for haplotypes of SNPs in each LD block, as well as for haplotypes of 2–5 SNPs in sliding windows using Family Based Association Test (FBAT; http://www.biostat.harvard.edu/~fbat/fbat.htm) [26] and PLINK (v1.07; http://pngu.mgh.harvard.edu/purcell/plink/) [27]. Bonferroni correction (http://www.quantitativeskills.com/sisa/calculations/bonfer.htm) was used to correct multiple comparisons. The overall empiric *P* value for the gene was generated from 10 000 permutations using Haploview as well (v4.2, http://www.broadinstitute.org/haploview/haploview) [24].

## Results

### Preliminary analysis

Among the 13 SNPs genotyped in and around *BMP4*, *rs2855527* was the only one with MAF<1% in Maryland (0.5%) and in Asian parents, and was excluded from further analysis. The remaining 12 SNPs were eligible because all were compatible with HWE (at p>0.01) and their genotyping call rates were >80% (83.6% to 100%).

The minor allele differed for three SNPs (*rs17563*, *rs10130587* and *rs2855530*) between Asian and Maryland parents among these 12 SNPs. MAF for *rs2761880* was low (3.9%) in Maryland parents compared to Asian parents (26.7%). MAFs for the other 8 SNPs were similar in Asian and Maryland parents, ranging from 42.3%–45.9% and 39.3%–46.1%, respectively (Table 2 and 3). The relative difference ratios for MAFs were less than 10% for these 8 SNPs (Table 3). LD patterns among these 12 SNPs were similar in Asian and Maryland parents (Fig S1). In Asian parents, the first 3 and last 7 SNPs formed LD blocks respectively; in Maryland parents, the first 2 and last 8 SNPs formed separate LD blocks. SNP *rs10130587* was an independent SNP in both Maryland and Asian parents.

### Association analysis

When deviation from strict Mendelian inheritance of individual SNPs was analyzed in Asian trios, three SNPs (*rs10130587*, *rs2738265* and *rs2761887*) showed nominally significant evidence of linkage and association (p<0.05) with NSCL/P. Significance held for *rs10130587* after strict Bonferroni correction (corrected p = 0.019). The overall empiric *P* value for *rs10130587* by Haploview was 0.018. Estimated odds ratio (OR) for carrying the apparent high-risk allele (MAF = 48.7%) at *rs10130587* was 1.61(95%CI = 1.20, 2.18). No significant evidence of linkage and association with NSCL/P was particularly evident for *rs10130587* in any of the three Asian groups or in a specific cleft type (cleft lip and palate or cleft lip only) in combined Asian population after Bonferroni correction (Table S2).

Sliding window haplotype analysis of these 12 SNPs in and around *BMP4* generated a total of 38 tests. All haplotypes including *rs10130587* were significantly associated with NSCL/P in combined Asian group. The Bonferroni corrected *P* value for the most significant haplotype (*rs2761880-rs10130587*) was not significant (0.0626) (Table S1). No significant linkage and association was seen in haplotype analysis of SNPs in each LD block (data not shown).

When 76 Maryland trios were analyzed, SNP *rs762642* in the second LD block gave nominally significant evidence of linkage and association (p = 0.0469), however, this result was not significant after Bonferroni correction (Table S1). In sliding window haplotype analysis, majority of the haplotypes including *rs762642* was nominally significant, but the most significant haplotype (*rs762643*- *rs762642*-*rs2761887*) was no longer significant after Bonferroni correction (Table S1). No significant linkage and association was seen in haplotype analysis of SNPs in each LD block (data not shown).

## Discussion

*BMP4* is located in 14q22-q23, and has 4 exons and 3 introns among which exons 3 and 4 encode protein (http://genome.ucsc.edu/cgi-bin). The protein encoded by this gene is a member of the bone morphogenetic protein family, part of the transforming growth factor-beta superfamily [28]. Animal experiments and genome wide linkage analysis in multiplex NSCL/P families repeatedly suggested *BMP4* play a potential role in risk for NSCL/P [13]–[18]. Several candidate gene association analyses [19]–[22] have also contributed evidence that *BMP4* is an important candidate gene for NSCL/P, although there has been no consistency across studies.

SNP *rs762642* is the most frequently reported SNP in literature on *BMP4* and its influence on NSCL/P [9]–[12], [19], [22]. One case-parent trio study from Chile tested the haplotype of *rs1957860-rs762642* and found significant evidence for linkage and association [19]. For individual SNP analysis, as with all previously published reports, no evidence was shown for *rs762642* either in our Asian or Maryland trios (after Bonferroni correction). And we did not find evidence in our sliding window haplotype analysis or in haplotype analysis for SNPs in identified LD block that included *rs762642* after Bonferroni correction.

SNP *rs17563* lies in exon 4 of *BMP4*, but evidence from association analysis with this marker has not been consistent. Two case-control studies conducted mainly in Han Chinese population [20], [21] and one resequencing study [29] using samples from six different populations showed evidence of association between *rs17563* and NSCL/P. However, evidence of linkage and association was not seen for *rs17563* in a case-parent trio study conducted in two Scandinavia populations using log-linear models [22]. Our negative results are consistent with Jugessur's analysis [22] although our sample size was quite limited.

Although no evidence of linkage and association was seen for *rs762642* and *rs17563* with NSCL/P, intriguing result was found for another SNP (*rs10130587*) in our Asian trios (which was significant after Bonferroni correction). SNP *rs10130587* was recently identified by the 1000 genomes program (http://www.ncbi.nlm.nih.gov/projects, 2011.12.8) with frequencies for allele *C* as 48.4% and 38.5% in combined Asian (Han Chinese in Beijing, Han Chinese South, and Japanese individuals) and CEPH samples separately (Table S3) [30]. Because MAF for this intronic SNP was high (48.7%) in our Asian parents, this marker should be informative and may be in LD with an unknown causal variant. The significant linkage and association observed in Asian NSCL/P trios was not replicated in our smaller sample of Maryland trios which also had a slightly lower MAF (40.8%) compared to our Asian founders. Because *rs10130587* was not genotyped in any previous candidate gene and GWAS studies [9]–[12], [19]–[22], our result can be considered an independent analysis although 52.9% (157 trios) of our sample [31] went into one GWAS [10]. It is possible that association for NSCL/P could have been identified with this SNP were it included in marker panels in previous association studies, including those failing to detect any evidence of linkage and association between *BMP4* and NSCL/P [9]–[12], [17], [22]. The arrays are always changing and it is likely that the version used for GWAS was optimized for Caucasians because most of the early HapMap sequencing was done with CEPH samples. Our results supported the published evidence for some association between *BMP4* and NSCL/P, and builds upon both experimental animal and linkage studies in humans.

## Supporting Information

Figure S1 Linkage disequilibrium as measured by *r^2^* in *BMP4* among parents of NSCL/P probands from Asian and Maryland trios. White: *r^2^* = 0. Shades of gray: 0<*r^2^*<1. Black: *r^2^* = 1. *BMP4*, Bone Morphogenetic Protein 4; NSCL/P, nonsyndromic cleft lip with or without cleft palate.
(DOC)

Table S1 Nominally significant tests from FBAT analysis for sliding window haplotypes (size 2–5 SNPs each) for 12 markers in *BMP4* in Asian and Maryland trios.
(DOC)

Table S2 TDT analysis for *rs10130587* in *BMP4* in trios from different Asian sites and by cleft type in combined Asian population.
(DOC)

Table S3 Frequency for allele *C* in *rs10130587* in *BMP4* by race group using data from 1000genomes.
(DOC)
