# Supplementary material for: BMP4 Was Associated with NSCL/P in an Asian Population
Source: PLoS One. 2012 Apr 13;7(4):e35347. doi: 10.1371/journal.pone.0035347 (PMC3325933; doi:10.1371/journal.pone.0035347)
Supplement: Figure S1 — Linkage disequilibrium as measured by r2 in BMP4 among parents of NSCL/P probands from Asian and Maryland trios. White: r2 = 0. Shades of gray: 0<r2<1. Black: r2 = 1. BMP4, Bone Morphogenetic Protein 4; NSCL/P, nonsyndromic cleft lip with or without cleft palate. (DOC) [file pone.0035347.s001.doc]

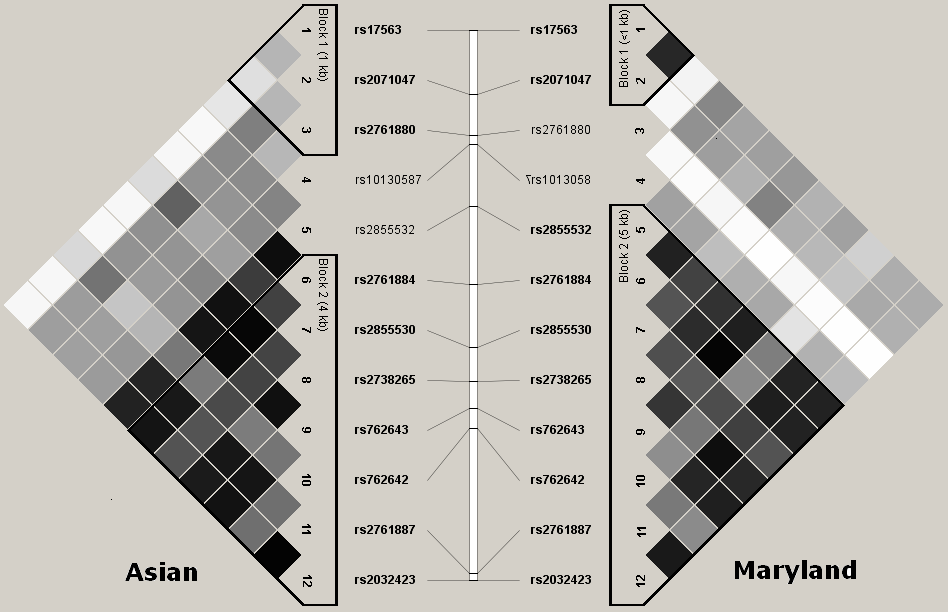


Fig S1. Linkage disequilibrium as measured by *r^2^* in *BMP4* among parents of NSCL/P probands from Asian and Maryland trios. White: *r^2^* = 0. Shades of gray: 0<*r^2^*<1. Black: *r^2^* = 1. *BMP4*, Bone Morphogenetic Protein 4; NSCL/P, nonsyndromic cleft lip with or without cleft palate.
