# Supplementary material for: BMP4 Was Associated with NSCL/P in an Asian Population
Source: PLoS One. 2012 Apr 13;7(4):e35347. doi: 10.1371/journal.pone.0035347 (PMC3325933; doi:10.1371/journal.pone.0035347)
Supplement: Table S1 — Nominally significant tests from FBAT analysis for sliding window haplotypes (size 2–5 SNPs each) for 12 markers in BMP4 in Asian and Maryland trios. (DOC) [file pone.0035347.s002.doc]

Table S1 Nominally significant tests from FBAT analysis for sliding window haplotypes (size 2-5 SNPs each) for 12 markers in *BMP4* in Asian and Maryland trios

| Haplotype | | | | | *P* value Asian | *P* value Maryland |
| --- | --- | --- | --- | --- | --- | --- |
| *rs2761880* | *rs10130587* | | | | 0.0017* | 0.5898 |
| *rs10130587* | *rs2855532* | | | | 0.0157 | 0.0868 |
| *rs762643* | *rs762642* | | | | 0.8270 | 0.0242 |
| *rs762642* | *rs2761887* | | | | 0.0371 | 0.0299 |
| *rs2761887* | *rs2032423* | | | | 0.0490 | 0.2070 |
| *rs2071047* | *rs2761880* | *rs10130587* | | | 0.0071 | 0.9024 |
| *rs2761880* | *rs10130587* | *rs2855532* | | | 0.0029 | 0.1304 |
| *rs10130587* | *rs2855532* | *rs2761884* | | | 0.0126 | 0.1082 |
| *rs2738265* | *rs762643* | *rs762642* | | | 0.8703 | 0.0176 |
| *rs762643* | *rs762642* | *rs2761887* | | | 0.3271 | 0.0104 |
| *rs762642* | *rs2761887* | *rs2032423* | | | 0.0671 | 0.0561 |
| *rs17563* | *rs2071047* | *rs2761880* | *rs10130587* | | 0.0196 | 0.8938 |
| *rs2071047* | *rs2761880* | *rs10130587* | *rs2855532* | | 0.0165 | 0.3249 |
| *rs2761880* | *rs10130587* | *rs2855532* | *rs2761884* | | 0.0046 | 0.1263 |
| *rs10130587* | *rs2855532* | *rs2761884* | *rs2855530* | | 0.0361 | 0.2194 |
| *rs2855530* | *rs2738265* | *rs762643* | *rs762642* | | 0.8879 | 0.0256 |
| *rs2738265* | *rs762643* | *rs762642* | *rs2761887* | | 0.5305 | 0.0183 |
| *rs762643* | *rs762642* | *rs2761887* | *rs2032423* | | 0.3280 | 0.0198 |
| *rs17563* | *rs2071047* | *rs2761880* | *rs10130587* | *rs2855532* | 0.0234 | 0.2705 |
| *rs2071047* | *rs2761880* | *rs10130587* | *rs2855532* | *rs2761884* | 0.0267 | 0.2408 |
| *rs2761880* | *rs10130587* | *rs2855532* | *rs2761884* | *rs2855530* | 0.0140 | 0.2152 |
| *rs10130587* | *rs2855532* | *rs2761884* | *rs2855530* | *rs2738265* | 0.0262 | 0.1963 |
| *rs2761884* | *rs2855530* | *rs2738265* | *rs762643* | *rs762642* | 0.8984 | 0.0249 |
| *rs2855530* | *rs2738265* | *rs762643* | *rs762642* | *rs2761887* | 0.6195 | 0.0257 |
| *rs2738265* | *rs762643* | *rs762642* | *rs2761887* | *rs2032423* | 0.5269 | 0.0321 |

*Bonferroni corrected P=0.0626 for this 2-SNP haplotype
