# Supplementary material for: BMP4 Was Associated with NSCL/P in an Asian Population
Source: PLoS One. 2012 Apr 13;7(4):e35347. doi: 10.1371/journal.pone.0035347 (PMC3325933; doi:10.1371/journal.pone.0035347)
Supplement: Table S2 — TDT analysis for rs10130587 in BMP4 in trios from different Asian sites and by cleft type in combined Asian population. (DOC) [file pone.0035347.s003.doc]

| Table S2 TDT analysis for *rs10130587* in *BMP4* in trios from different Asian sites  and by cleft type in combined Asian population | | | | | | |
| --- | --- | --- | --- | --- | --- | --- |
| Site/cleft type | Risk allele freq (%) | FAM* | T | NT | OR (95%CI) | *P* Value |
| Site |  |  |  |  |  |  |
| Taiwan | 47.7 | 96 | 77 | 52 | 1.48 (1.04,2.11) | 0.0277 |
| Singapore | 56.8 | 17 | 16 | 5 | 3.20 (1.17,8.73) | 0.0164 |
| Korea | 45.9 | 26 | 20 | 13 | 1.54 (0.77,3.09) | 0.2230 |
| Asian combined | 48.7 | 139 | 113 | 70 | 1.61 (1.20,2.18) | 0.0016 |
| Cleft type |  |  |  |  |  |  |
| Nonsyndromic cleft lip only | 45.2 | 31 | 27 | 13 | 2.08 (1.07,4.03) | 0.0269 |
| Nonsyndromic cleft lip and palate | 49.2 | 108 | 86 | 57 | 1.51 (1.08,2.11) | 0.0153 |
| FAM*: number of informative families, T: number of transmitted alleles, NT: number of un-transmitted alleles | | | | | | |
