# Supplementary material for: BMP4 Was Associated with NSCL/P in an Asian Population
Source: PLoS One. 2012 Apr 13;7(4):e35347. doi: 10.1371/journal.pone.0035347 (PMC3325933; doi:10.1371/journal.pone.0035347)
Supplement: Table S3 — Frequency for allele C in rs10130587 in BMP4 by race group using data from 1000genomes. (DOC) [file pone.0035347.s004.doc]

| Table S3 Frequency for allele *C* in *rs10130587* in *BMP4* | | | |
| --- | --- | --- | --- |
| by race group using data from 1000genomes | | | |
| Race group | population | # | allele *C* |
|  |  | subjects | freq(%) |
| Asian | Han Chinese in Beijing | 97 | 45.9 |
|  | Han Chinese South | 100 | 45.0 |
|  | Japanese individuals | 89 | 55.1 |
| Subtotal |  | 286 | 48.4 |
| Caucasion | CEPH individuals | 87 | 38.5 |
|  | British individuals from England and Scotland | 89 | 36.0 |
|  | Toscan individuals | 98 | 46.9 |
|  | Finnish individuals from Finland | 93 | 34.4 |
| Subtotal |  | 367 | 39.1 |
| African | Yoruba individuals | 88 | 55.1 |
|  | Luhya individuals | 97 | 63.4 |
|  | African ancestry individuals from SW US | 61 | 53.3 |
| Subtotal |  | 246 | 57.9 |
| Mixture | Mexican individuals from LA California | 66 | 36.4 |
|  | Puerto Rican in Puerto Rico | 55 | 50.9 |
|  | Colombian in Medellin, Colombia | 60 | 40.0 |
| Iberian | Iberian populations in Spain | 14 | 46.4 |
| Total |  | 1094 | 46.3 |
